# Tea Polyphenols Attenuate Methamphetamine-Induced Neuronal Damage in PC12 Cells by Alleviating Oxidative Stress and Promoting DNA Repair

**DOI:** 10.3389/fphys.2019.01450

**Published:** 2019-12-05

**Authors:** Qin Ru, Qi Xiong, Xiang Tian, Lin Chen, Mei Zhou, Yi Li, Chaoying Li

**Affiliations:** ^1^Wuhan Institutes of Biomedical Sciences, School of Medicine, Jianghan University, Wuhan, China; ^2^Department of Psychiatry, Wuhan Mental Health Center, Wuhan, China

**Keywords:** tea polyphenols, methamphetamine, apoptosis, DNA damage, oxidative stress

## Abstract

DNA integrity plays a crucial role in cell survival. Methamphetamine (METH) is an illegal psychoactive substance that is abused worldwide, and repeated exposure to METH could form mass free radicals and induce neuronal apoptosis. It has been reported that free radicals generated by METH treatment can oxidize DNA and hence produce strand breaks, but whether oxidative DNA damage is involved in the neurotoxicity caused by METH remains unclear. Tea polyphenols exert bioactivities through antioxidant-related mechanisms. However, the potential neuroprotective effect of tea polyphenols on METH-induced nerve cell damage and the underlying mechanism remain to be clarified. In this study, oxidative stress, DNA damage, and cell apoptosis were increased after METH exposure, and the expressions of DNA repair-associated proteins, including the phosphorylation of ataxia telangiectasia mutant (p-ATM) and checkpoint kinase 2 (p-Chk2), significantly declined in PC12 cells after high-dose or long-time METH treatment. Additionally, tea polyphenols could protect PC12 cells against METH-induced cell viability loss, reactive oxide species and nitric oxide production, and mitochondrial dysfunction and suppress METH-induced apoptosis. Furthermore, tea polyphenols could increase the antioxidant capacities and expressions of p-ATM and p-Chk2 and then attenuate DNA damage *via* activating the DNA repair signaling pathway. These findings indicate that METH is likely to induce neurotoxicity by inducing DNA damage, which can be reversed by tea polyphenols. Supplementation with tea polyphenols could be an effective nutritional prevention strategy for METH-induced neurotoxicity and neurodegenerative disease.

## Introduction

The synthetic central stimulant methamphetamine (METH) is widely abused in the world. Clinical toxicology surveys have shown that METH can induce neuronal damage in abusers (Gold et al., 2009; O’Dell and Marshall, 2014; Ren et al., 2016). In line with these clinical reports, numerous animal studies have revealed that METH can induce long-term damage to dopaminergic neurons and cause cell apoptosis (Li et al., 2017; Dang et al., 2018). The neurotoxicity of METH is mainly believed to be dependent on its structural similarity to dopamine (DA), which makes it easier for it to enter dopaminergic neurons *via* the DA transporter (DAT) and causes DA to be over-released into the cytoplasm, where DA can undergo auto-oxidation rapidly to form a large number of toxic materials such as superoxide radicals, resulting in oxidative stress, decreased mitochondrial membrane potential (ΔΨm), and neuronal apoptosis (Krasnova and Cadet, 2009). METH treatment may also lead to a decline in superoxide dismutase (SOD) and glutathione peroxidase activities, with increased lipid peroxidation and levels of reactive oxygen species (ROS) (Qie et al., 2017). Pretreatment with antioxidants such as N-acetylcysteine has been shown to exert neuroprotection against the nerve damage caused by METH (Nakagawa et al., 2018). However, little is known concerning how METH impairs adaptation to cellular stresses such as oxidant injury and can thus cause cellular dysfunction leading to disease.

Genome integrity is important for cell survival. DNA damage is closely related to the growth status and function of cells, so nerve damage caused by METH may be related to DNA damage. Based on the generally accepted theory, highly conserved DNA repair system including ataxia telangiectasia mutant (ATM) and checkpoint kinase 2 (Chk2) can deal with both exogenous and endogenous DNA damage under normal conditions, resulting in damage at low homeostasis levels compatible with normal cellular function (Terabayashi and Hanada, 2018). However, endogenous damage cannot be repaired in a timely manner under the condition of DNA repair deficiency and keeps accumulating over time, leading to unscheduled alterations in the genome or instability, which can induce cell damage or apoptosis (Mirza-Aghazadeh-Attari et al., 2018). The neurotoxicity induced by the accumulation of DNA damage has been widely reported in neurodegenerative disease (Fernandez-Bertolez et al., 2018; Wu et al., 2018). For instance, alcohol abuse may significantly increase the level of ROS, which leads to DNA damage and may trigger apoptosis *via* activation of the mitochondrial pathway (Fowler et al., 2012; Kotova et al., 2013). Repeated exposure to METH could form large amounts of free radicals and causes DNA oxidation and strand breaks (Johnson et al., 2015). Therefore, we speculated that DNA damage may be an important cause of neurotoxicity induced by METH and that free radicals may be involved in DNA damage and apoptosis, while reducing the levels of free radicals could partially inhibit METH-induced neuronal DNA damage and apoptosis.

Tea polyphenols are natural compounds extracted from tea leaves and show good antioxidant capacities both *in vitro* and *in vivo* (Mao et al., 2017; Qi et al., 2017a, 2018). However, there have been few reports regarding whether tea polyphenols have a protective effect on METH-induced neuronal damage. Therefore, the purpose of the current research was to study whether tea polyphenols could alleviate apoptosis induced by METH through the inhibition of oxidative stress and DNA damage in dopaminergic neurons. For this purpose, we determined cell survival rates, apoptotic rates, ΔΨm, ROS production, oxidative enzyme activities, nitric oxide (NO) production, and expressions of DNA damage and repair-related proteins in rat adrenal pheochromocytoma cells (PC12). PC12 cells were selected because they can synthesize and store DA, and they have many biochemical mechanisms related to dopaminergic cells (Greene and Tischler, 1976; Li et al., 2017). The results of this study demonstrate that METH exposure can increase oxidative stress and DNA damage and that tea polyphenols may be considered an effective protective substance to mitigate the DNA damage and apoptosis caused by METH in future clinical practice.

## Materials and Methods

### Chemicals and Drug Preparations

Methamphetamine (METH) was provided by the Hubei Provincial Public Security Department. Tea polyphenols were purchased from Beijing Yihua Tongbiao Technology Co. Ltd. (tea polyphenol purity >98%, including catechin content >70%, epigallocatechin gallate content >40%; Beijing, China). 3-(4,5-Dimethylthiazol-2-yl)-2,5-diphenyltetrazolium bromide (MTT) and 2′,7′-dichlorodihydrofluorescein diacetate (DCFH-DA) were purchased from Sigma-Aldrich, Inc. (St Louis, USA). Fetal bovine serum (FBS) and RPMI-1640 medium were purchased from Thermo Fisher Scientific (Carlsbad, USA). The Muse MitoPotential Kit, Muse Multi-Color DNA Damage kit, and Muse Annexin V & Dead Cell Kit were procured from Millipore Corporation (Darmstadt, Germany). The lactate dehydrogenase (LDH) cytotoxicity assay kit, reduced glutathione (GSH) assay kit, total superoxide dismutase (T-SOD) assay kit, and malondialdehyde (MDA) assay kit were procured from Nanjing Jiancheng Bioengineering Institute (Nanjing, China). The NO and ΔΨm detection kit were obtained from Beyotime Biotechnology (Haimen, China). The RIPA lysis buffer, phosphatase inhibitors, and protease inhibitor cocktail were purchased from Boster Biological Technology Co. Ltd. (Wuhan, China). Antibodies against cleaved caspase-3, phospho-ATM (p-ATM), phospho-Histone H2AX (γ-H2AX), and phospho-Chk2 (p-Chk2) were purchased from Cell Signaling Technology, Inc. (Beverly, USA). All other chemicals were of analytical grade.

### Cell Culture

Rat adrenal pheochromocytoma cells (PC12, high differentiation) were provided by the cell bank of the Chinese Academy of Sciences (Shanghai, China) and cultured in RPMI-1640 medium containing 10% FBS (complete medium). Cells were passaged every 3 days to maintain exponential growth.

### Cell Proliferation Experiment

Cell proliferation was examined by using MTT assay. Cells were seeded in 96-well plates and incubated overnight. MTT solution (final concentration of 0.5 mg/ml) was added after treatment with different substances, and incubation was continued for 4 h. The culture medium was then discarded, and DMSO was added into each well. Subsequently, a microplate reader (Thermo Scientific, USA) was used to measure the absorbance at 570 nm.

### Lactate Dehydrogenase Release Assay

The cytotoxicity of cells exposed to different treatments was determined by LDH activity in culture medium. After being treated with different substances, the cultural supernatant of each well was transferred as the measured group. According to the instructions of the manufacturer, the blank group, control group, and standard group were also prepared. Finally, optical density (OD) was measured at 450 nm using the microplate reader. The activity of LDH was calculated according to the following formula:

LDHactivityUL=ODmeasured–ODcontrolODstandard−ODblank×0.2mmol/L×1,000

### Cell Apoptosis Detection

Cell apoptosis was detected by flow cytometry. After treatment, cells were harvested and resuspended in a mixture of complete medium and Annexin V and Dead cell reagent. The mixture was incubated with gentle oscillation at 25°C for 20 min. In apoptotic cells, the membrane phospholipid phosphatidylserine (PS) is translocated from the inner to the outer leaflet of the plasma membrane, thereby exposing PS to the external cellular environment. Annexin V has a high affinity for PS and binds to cells with exposed PS. In Annexin V and Dead cell reagent, Annexin V is conjugated to fluorescein (FITC). This format retains its high affinity for PS and thus serves as a sensitive probe for flow cytometric analysis of cells that are undergoing apoptosis. In addition, the reagent includes 7-Amino-Actinomycin (7-AAD), which can bind tightly to the nucleic acids in cells and is impermeant to live cells and early apoptotic cells but stains dead cells and late apoptotic cells. The percentage of apoptotic cells was quantified with flow cytometry (Muse Cell Analyzer, Germany).

### Analysis of Mitochondrial Membrane Potential

The changes in ΔΨm were detected by using JC-1 in PC-12 cells treated with METH with or without tea polyphenols. JC-1 is a cationic dye that accumulates in energized mitochondria. JC-1 is predominantly a monomer that yields green fluorescence with an emission of 530 ± 15 nm at low ΔΨm and aggregates, yielding a red-colored emission (590 ± 17.5 nm), at high ΔΨm. After being treated, cells were stained with JC-1 working solution, rinsed twice with ice-cold staining buffer, resuspended in complete medium, and immediately examined with a fluorescent microscope (IX71, Olympus, Japan). The excitation wavelength of JC-1 monomers was 488 nm, and the emission wavelength was 535 nm. The excitation wavelength of JC-1 aggregates was 525 nm, and the emission wavelength was 595 nm.

The proportion of cells in which ΔΨm had declined was measured by using a Muse MitoPotential Kit. After being treated, cells were harvested and suspended in assay buffer. Changes in ΔΨm were then evaluated according to the instructions of the manufacturer.

### DNA Damage Detection

Alkaline Comet Assay was applied to detect DNA strand breaks. Cells were suspended in low-melting agarose, plated on pre-coated microscope slides, and lysed in pre-chilled lysis solution for 1 h at 4°C. After incubation in alkaline buffer for 20 min, cells were electrophoresed for 15 min at 25 V, soaked in neutralization buffer for 5 min, and dyed with ethidium bromide in the dark. Images were analyzed using a fluorescent microscope 20 min later, and comets were analyzed with the Comet Assay Software Project. The percentage of DNA in the tail was used to reflect the extent of DNA damage.

A Muse Multicolor DNA Damage Kit was also used to investigate the DNA damage. After being treated, cells were harvested, washed with cold PBS, and fixed for 10 min on ice. The percentage of DNA-damaged cells was quantified after being permeabilized according to the manufacturer’s instructions.

### Measurement of Nitric Oxide Release

Quantitative determination of nitrite ions was applied as an indirect method for determining the level of NO. In simple terms, cells were seeded and incubated for 24 h. After exposed to different substances, cell culture mediums were collected to analyze the release of NO, following the manufacturer’s instructions.

### Detection of Reactive Oxygen Species

DCFH-DA can be oxidized by ROS into 2′,7′-dichlorofluorescin (DCF) after entering cells, so the intensity of DCF can represent the level of intracellular ROS. After different treatments, cells were suspended in DCFH-DA solution, and the cell suspensions were incubated at 37°C for 20 min. PBS was then used to resuspend the cells, and the intensity of DCF was measured with a fluorescent microplate reader (excitation 485 nm, emission 500 nm).

### Determination of Antioxidant System and Lipid Peroxidation

The oxidative stress induced by METH and the protection from tea polyphenols were assessed by using the oxidative enzyme system (SOD, GSH, and MDA) to examine the oxygen reactivity of PC12 cells. After treatment with different substances, cells were harvested, resuspended, sonicated, and centrifuged for 15 min at 4,000 rpm. Subsequently, supernatants were individually used to measure the activities of the antioxidases SOD and GSH using corresponding diagnostic kits. The concentration of MDA in supernatants, which could express the degree of lipid peroxidation, was also determined according to the instructions of the manufacturer.

### Western Blotting

RIPA buffer supplemented with phosphatase inhibitors and cocktail was used to lyse PC12 cells, and lysed cells were centrifuged for 15 min at 12,000 rpm to gather the supernatant. Stain-Free Gels (Bio-Rad) were used to separate proteins, and isolated proteins were electroblotted onto polyvinylidene difluoride (PVDF) membrane (Millipore). After blocking, the PVDF membrane was incubated with primary antibodies (anti-cleaved caspase-3, anti-γ-H2AX anti-p-ATM, and anti-p-Chk2) overnight at 4°C, followed by a horseradish-peroxidase-conjugated secondary antibody. Finally, the PVDF membrane was incubated with ECL substrate (Thermo Fisher Scientific Inc.) and scanned with a ChemiDoc Touch Imager (Bio-Rad). The results of Western Blotting were analyzed with Image J. All gels were imaged after electrophoresis. It has been reported in previous studies that normalization of samples to total protein density values is more reliable than normalization to individual protein levels (Vegh et al., 2014).

### Statistical Analysis

All data are shown as means ± SEM. Differences among groups were calculated by one-way ANOVA using SPSS 20.0 software, and Tukey’s HSD was applied as a post-hoc test. *p* < 0.05 was considered statistically significant.

## Results

### Effect of Methamphetamine on Cell Viabilities in PC12 Cells

**Figure 1:**
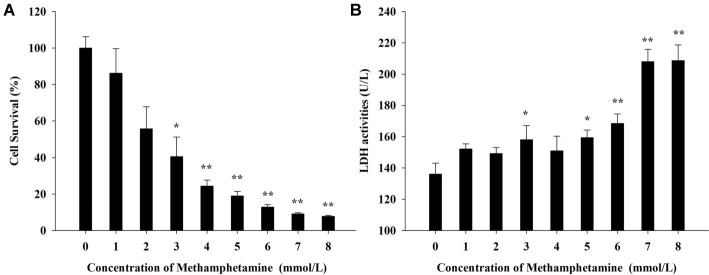
Effect of methamphetamine on the viability in PC12 cells. **(A)** After PC12 cells were treated with different concentrations of methamphetamine for 24 h, the cell viability was measured by MTT assay. **(B)** After PC12 cells were treated with different concentrations of methamphetamine for 24 h, the activities of LDH in the supernatant of culture medium were detected. Data are presented as mean ± SEM; ^*^p < 0.05 and ^**^p < 0.01 versus the control group.

Both MTT assay and LDH cytotoxicity assay were used to measure the effect of METH on the viabilities of PC12 cells. Figure 1A shows that METH could significantly inhibit the proliferation of PC12 cells in MTT assay and that exposure to 3 mmol/L METH caused a 40.5 ± 5.3% reduction in the number of viable PC12 cells (*p* < 0.01). Meanwhile, the activities of LDH progressively increased by 23.6, 29.2, and 43.1%, respectively, after incubation with 3, 5, and 7 mmol/L METH for 24 h compared with the control group (*p* < 0.05, Figure 1B). Collectively, these results suggested that METH exposure could induce significant neurotoxic effects in PC12 cells *in vitro*.

### Effect of Methamphetamine on Nitric Oxide and Reactive Oxygen Species Levels in PC12 Cells

**Figure 2:**
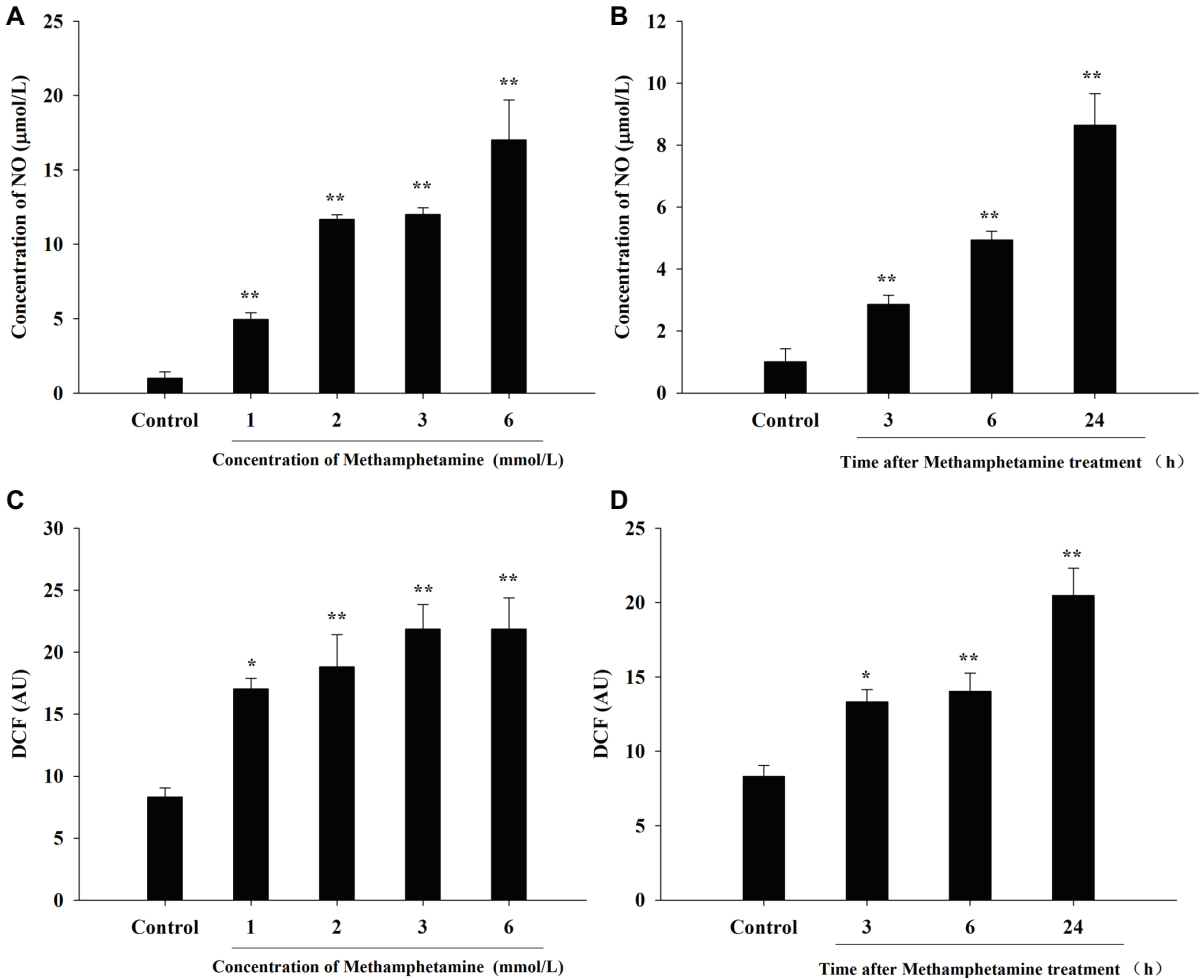
Effect of methamphetamine on NO and ROS production in PC12 cells. PC12 cells were treated with 1–6 mmol/L METH for 24 h **(A,C)** or with 3.0 mmol/L METH for 3–24 h, as indicated **(B,D)**. The supernatant from each group was collected to determine the production of NO **(A,B)**. DCFH-DA was incubated with cells for 1 h in a CO_2_ incubator, and then the fluorescent signal was obtained to evaluate the intracellular ROS level in different groups **(C,D)**. Data are presented as mean ± SEM; ^*^p < 0.05 and ^**^p < 0.01 versus the control group.

To investigate whether oxidative stress is involved in the cytotoxic effect of METH, the production of NO and ROS was examined (Figure 2). The results showed that levels of NO and ROS were remarkably increased after METH exposure compared with the control group. For instance, exposure to 1.0 mmol/L METH for 24 h increased NO- and ROS-production by 4.96-fold and 2.05-fold, respectively (*p* < 0.05), and these increases reached 12.00-fold and 2.63-fold, respectively, when the dose of METH was 3.0 mmol/L (*p* < 0.01). The levels of NO and ROS production were 2.83-fold and 1.60-fold higher respectively after METH (3.0 mmol/L) treatment for 3 h (*p* < 0.05), and these effects increased gradually over time. These results indicated that METH induced significant oxidative stress in PC12 cells.

### Effect of Methamphetamine on the ΔΨm in PC12 Cells

**Figure 3:**
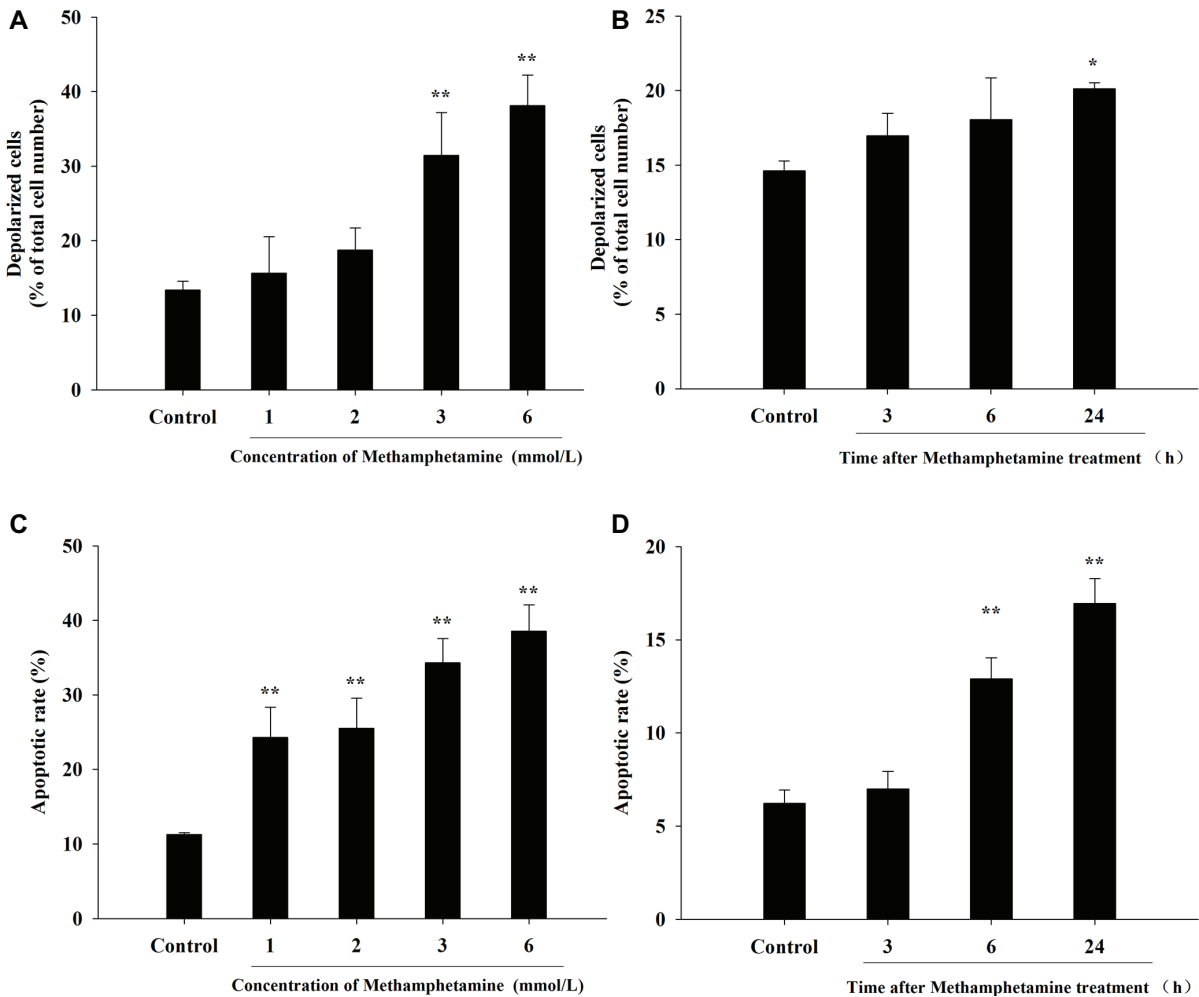
Effect of methamphetamine on mitochondrial membrane potential and apoptosis in PC12 cells. PC12 cells were treated with 1–6 mmol/L METH for 24 h **(A,C)** or with 3.0 mmol/L METH for 3–24, as indicated **(B,D)**. Mitochondrial membrane potentials in different groups were analyzed by Muse MitoPotential assay **(A,B)**. Cell apoptosis was analyzed by Muse Annexin V & Dead Cell Assay **(C,D)**. Data are presented as mean ± SEM; ^*^p < 0.05 and ^**^p < 0.01 versus the control group.

The loss of ΔΨm, an indication of mitochondrial function, is one of the important indicators of apoptosis. Compared with the control group, METH induced a significant increase in total mitochondrial depolarization (Figures 3A,B, *p* < 0.05). For instance, compared to the control group, the proportion of depolarized cells in 3 mmol/L and 6 mmol/L METH-treated cells were increased significantly by 2.35-fold and 2.85-fold, respectively, after 24-h exposure (*p* < 0.05).

### Effect of Methamphetamine on Cell Apoptosis in PC12 Cells

**Figure 4:**
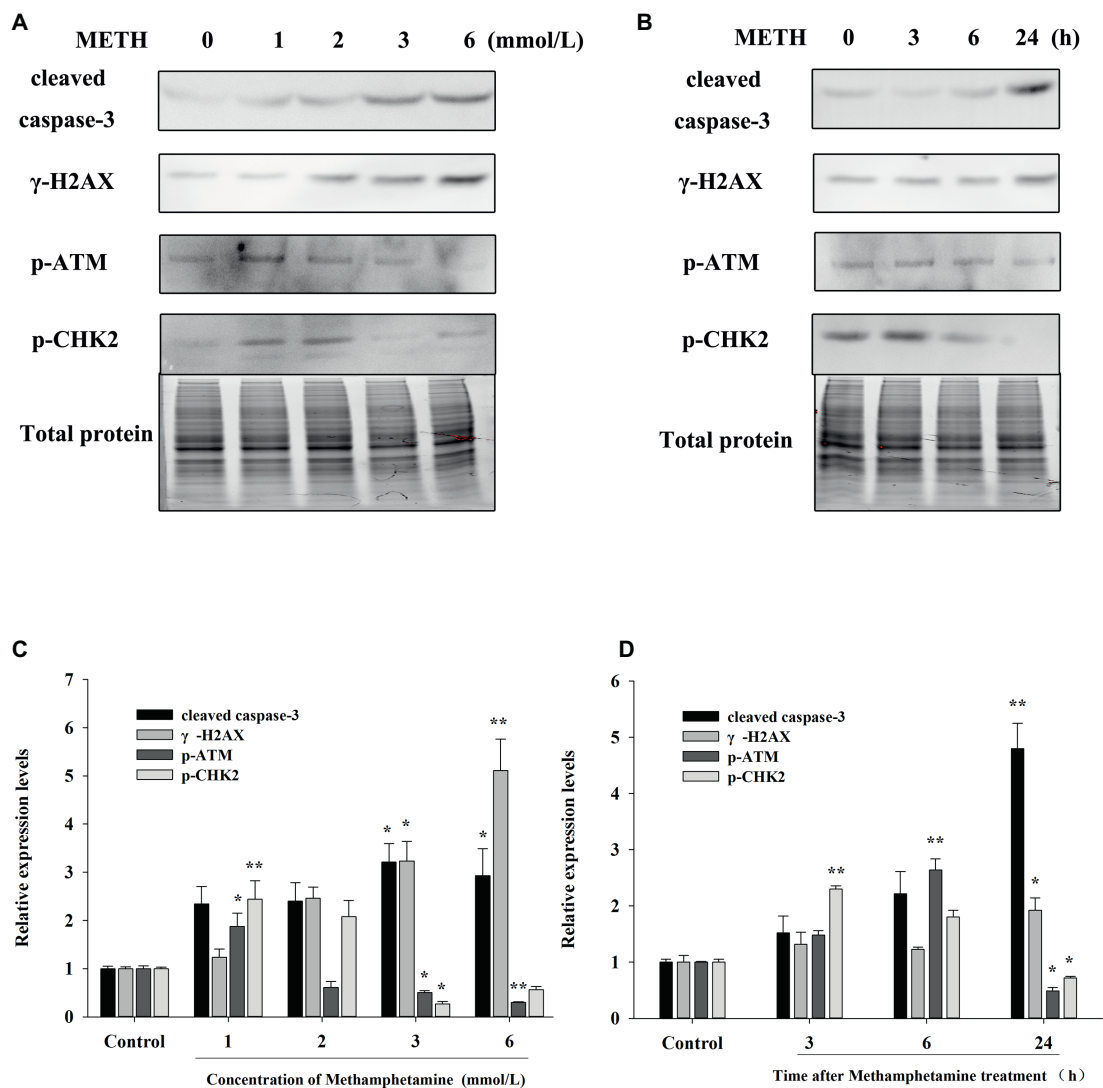
Effect of methamphetamine on apoptosis and DNA damage in PC12 cells. **(A)** PC12 cells were treated with 1–6 mmol/L METH for 24 h, and the expressions of cleaved caspase-3, γ-H2AX, p-ATM, and p-CHK2 were detected using Western Blot; total protein was used as a loading control. Densitometric analysis of the blots is shown in **(B)**. **(C)** PC12 cells were treated with 3.0 mmol/L METH for 3–24 h, and the expressions of cleaved caspase-3, γ-H2AX, p-ATM, and p-CHK2 were detected using Western Blot; total protein was used as a loading control. Densitometric analysis of the blots is shown in **(D)**. Data are presented as mean ± SEM, *p < 0.05 and **p < 0.01 versus the control group.

The flow cytometry results showed that METH treatment could remarkably increase cell apoptosis, and the apoptosis rate increased with an increase in the concentration and incubation time (Figures 3C,D). For example, after exposure to METH (1 mmol/L) for 24 h, the rate of apoptotic cells increased 2.2-fold (*p* < 0.01), and this increase reached 3.4-fold when the dose of METH was 6 mmol/L (*p* < 0.01). In addition, the rate of apoptotic cells was increased to 2.1-fold higher after 3 mmol/L METH treatment for 6 h (*p* < 0.01), and this effect increased gradually over time. As shown in Figure 4, the protein levels of cleaved caspase-3 in METH-treated groups were also significantly higher than that of the control group (*p* < 0.05).

### Effect of Methamphetamine on DNA Damage in PC12 Cells

The expressions of DNA damage and repair-related protein markers in PC12 cells after METH treatment were determined to evaluate the influence of METH on DNA damage. As showed in Figure 4, METH exposure greatly increased the expression of γ-H2AX. For instance, exposure to METH (2.0 mmol/L) for 24 h increased the expression of γ-H2AX 2.45-fold (*p* < 0.05), and this increase reached 5.11-fold when the dose of METH was 6.0 mmol/L (*p* < 0.01). In addition, the expression of γ-H2AX increased gradually with an increase in meth concentration (*p* < 0.05). These findings indicated that METH could induce DNA damage in PC12 cells.

The results in Figure 4 also show that low-dose or short-time METH treatment could significantly increase the expressions of p-ATM and p-Chk2, while high-dose or long-time METH treatment could reduce their expression levels (Figure 4). For instance, exposure to 1.0 mmol/L METH for 24 h and exposure to 3.0 mmol/L METH for 3 h could significantly increase the protein levels of p-ATM and p-Chk2 (*p* < 0.05), while their expressions were decreased substantially compared with the control group by 24-h exposure to 3.0 mmol/L METH (*p* < 0.05). These results showed that METH could induce significant DNA damage and inhibit the activation of the DNA repair system in PC12 cells, and the cell apoptosis caused by METH may be related to the oxidative stress and DNA damage that it induces.

### Effect of Tea Polyphenols on the Cytotoxicity of PC12 Cells Caused by Methamphetamine

**Figure 5:**
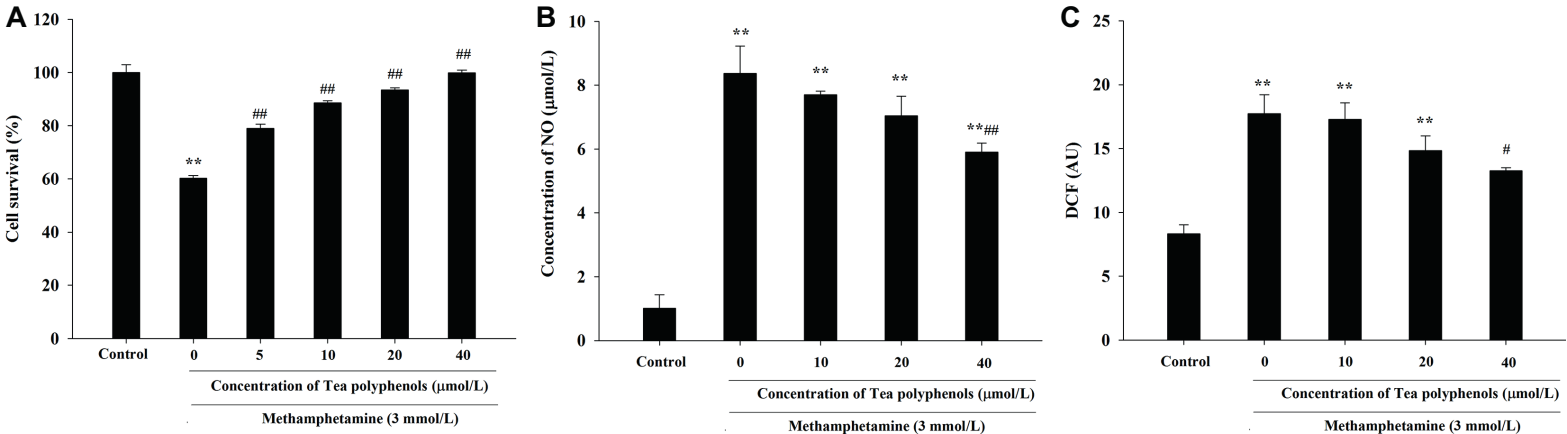
Intervention effects of tea polyphenols on viability and oxidative stress in PC12 neuronal cells. PC12 cells were treated with METH (3.0 mmol/L) with or without treatment with tea polyphenols (5, 10, 20, and 40 μmol/L) for 24 h. **(A)** Cell viability was measured by MTT assay. **(B)** The supernatant from each group was collected to determine the production of NO. **(C)** DCFH-DA was incubated with cells for 1 h in a CO_2_ incubator, and then the fluorescent signal was obtained to evaluate the intracellular ROS level in different groups. Data are presented as mean ± SEM; ^**^p < 0.01 versus the control group; ^#^*p* < 0.05 and ^##^*p* < 0.01 versus the METH group.

As shown in Figure 5A, the cell survival rate of METH-treated group was significantly lower than that of the control group (*p* < 0.01), and the survival rates of groups treated with different doses of tea polyphenols (5, 10, 20, and 40 μmol/L; *p* < 0.05) were increased significantly compared with the METH group. For instance, the cell survival rate of the 40-μmol/L tea polyphenols group was 99.93 ± 0.91%, while that of the METH group was 60.23 ± 0.97% (*p* < 0.01).

### Effect of Tea Polyphenols on Nitric Oxide and Reactive Oxygen Species Production After Methamphetamine Exposure in PC 12 Cells

As shown in Figure 5B, NO production was increased significantly in the METH group compared with the control group (*p* < 0.01). When the concentration of tea polyphenols increased to 20 μmol/L, the contents of NO were decreased significantly compared with the METH group (*p* < 0.05). As shown in Figure 5C, METH also significantly promoted the generation of ROS (*p* < 0.01). Additionally, with incremental increases in the dose of tea polyphenols, the levels of ROS gradually decreased, and 40 μmol/L tea polyphenols significantly decreased the ROS production in comparison with the METH group (*p* < 0.05).

### Effect of Tea Polyphenols on the Antioxidant System and Lipid Peroxidation After METH Exposure in PC12 Cells

**Figure 6:**
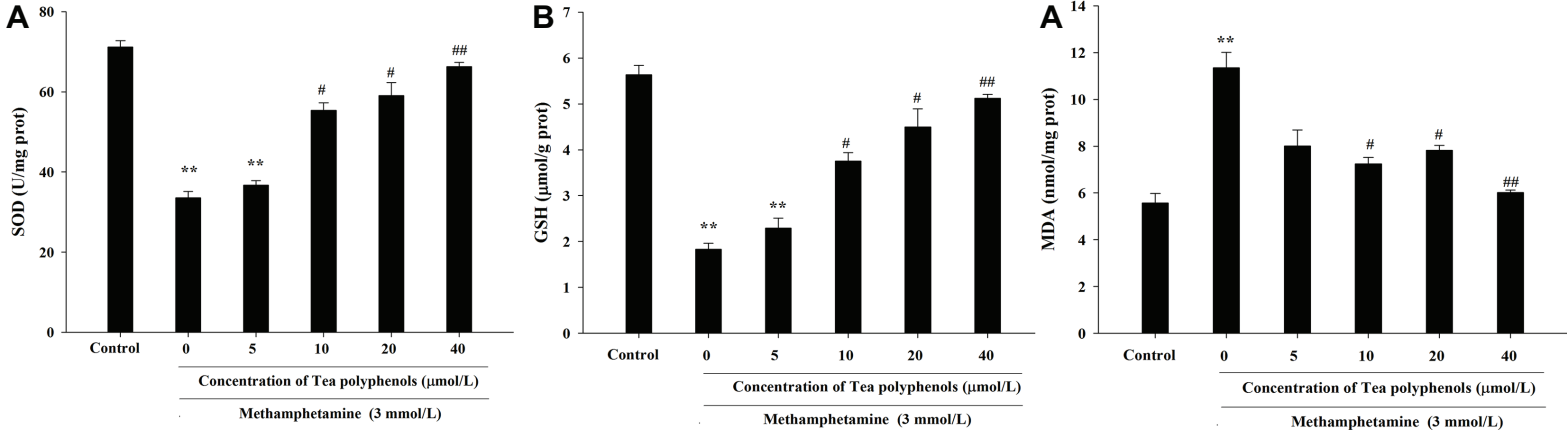
Intervention effects of tea polyphenols on the oxidative enzyme system in PC12 neuronal cells. PC12 cells were treated with METH (3.0 mmol/L) with or without treatment with tea polyphenols (5, 10, 20, and 40 μmol/L) for 24 h. Cell samples were collected for measurements of the levels of SOD **(A)**, GSH **(B)**, and MDA **(C)** using corresponding commercial detection kits. Data are presented as mean ± SEM; ^**^p < 0.01 versus the control group; ^#^*p* < 0.05 and ^##^*p* < 0.01 versus the METH group.

Figure 6 presents the data from the SOD, GSH, and MDA detection experiments. Compared with the control group, SOD activities in the METH group were significantly reduced in PC12 cells (71.21 ± 1.52 vs. 33.51 ± 1.59, *p* < 0.01), and 10, 20, and 40 μmol/L of tea polyphenols significantly increased SOD activities compared with the METH group (Figure 6A, *p* < 0.05). Additionally, METH significantly decreased GSH levels compared with the control group (1.83 ± 0.13 vs. 5.64 ± 0.21, *p* < 0.01), and 10, 20, and 40 μmol/L of tea polyphenols significantly increased GSH levels (Figure 6B, *p* < 0.05). Finally, the MDA contents were greatly increased after METH treatment (5.57 ± 0.42 vs. 11.36 ± 0.65, *p* < 0.01) compared with the control group, and all doses of tea polyphenols decreased the MDA contents significantly (8.00 ± 0.68, 7.23 ± 0.29, 7.82 ± 0.21, 6.02 ± 0.10 vs. 11.36 ± 0.65, Figure 6C, *p* < 0.05).

### Effect of Tea Polyphenols on ΔΨM and Cell Apoptosis After Methamphetamine Exposure in PC 12 Cells

**Figure 7:**
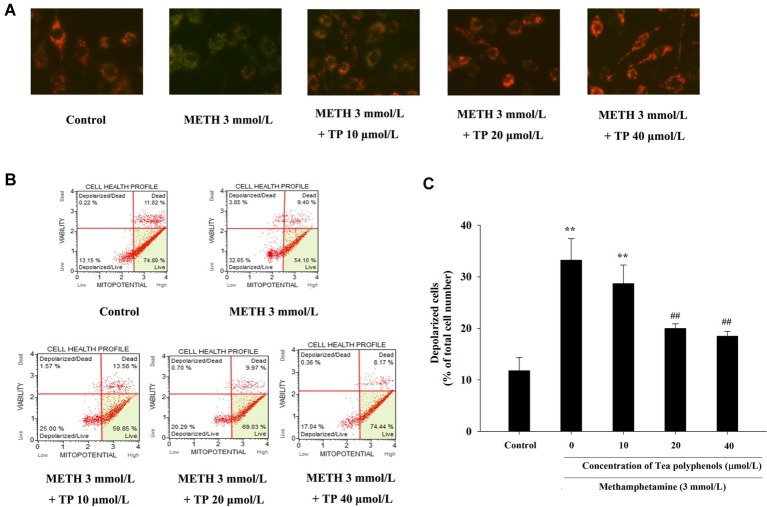
Effect of tea polyphenols on mitochondrial membrane potential in PC 12 cells after METH exposure. PC12 cells were treated with METH (3.0 mmol/L) with or without treatment with tea polyphenols (5, 10, 20, and 40 μmol/L) for 24 h. **(A)** The mitochondrial membrane potential was examined by JC-1 staining (×200). The mitochondrial membrane potential in PC 12 cells was also analyzed by Muse MitoPotential assay. Representative dot plots in the live, depolarized/live, depolarized/dead, and dead phases are shown in the left panel **(B)**, and the mean percentage of depolarized cells is expressed in a histogram in the right panel **(C)**. Data are presented as mean ± SEM; ^**^p < 0.01 versus the control group; ^##^*p* < 0.01 versus the METH group.

JC-1 assay and flow cytometry were both used to measure mitochondrial depolarization, which occurs in the early phase of apoptosis. In the JC-1 assay, decreased red fluorescence and increased green fluorescence represented decreased ΔΨm in mitochondria. As shown in Figure 7A, the increased number of green-stained cells indicated that METH had a strong pro-apoptotic effect on PC 12 cells, and different concentrations of tea polyphenols could effectively inhibit the METH-induced decrease in ΔΨm. Muse MitoPotential assay was applied to further detect the proportion of cells with decreased membrane potential (Figure 7B). Figure 7C shows that METH could significantly increase the rate of mitochondrial depolarized cells in comparison with the control group (*p* < 0.01), indicating that a remarkable dissipation of ΔΨm was induced by METH. In addition, 20 and 40 μmol/L tea polyphenols could greatly reduce the total depolarization of mitochondria compared with the METH group (*p* < 0.01).

**Figure 8:**
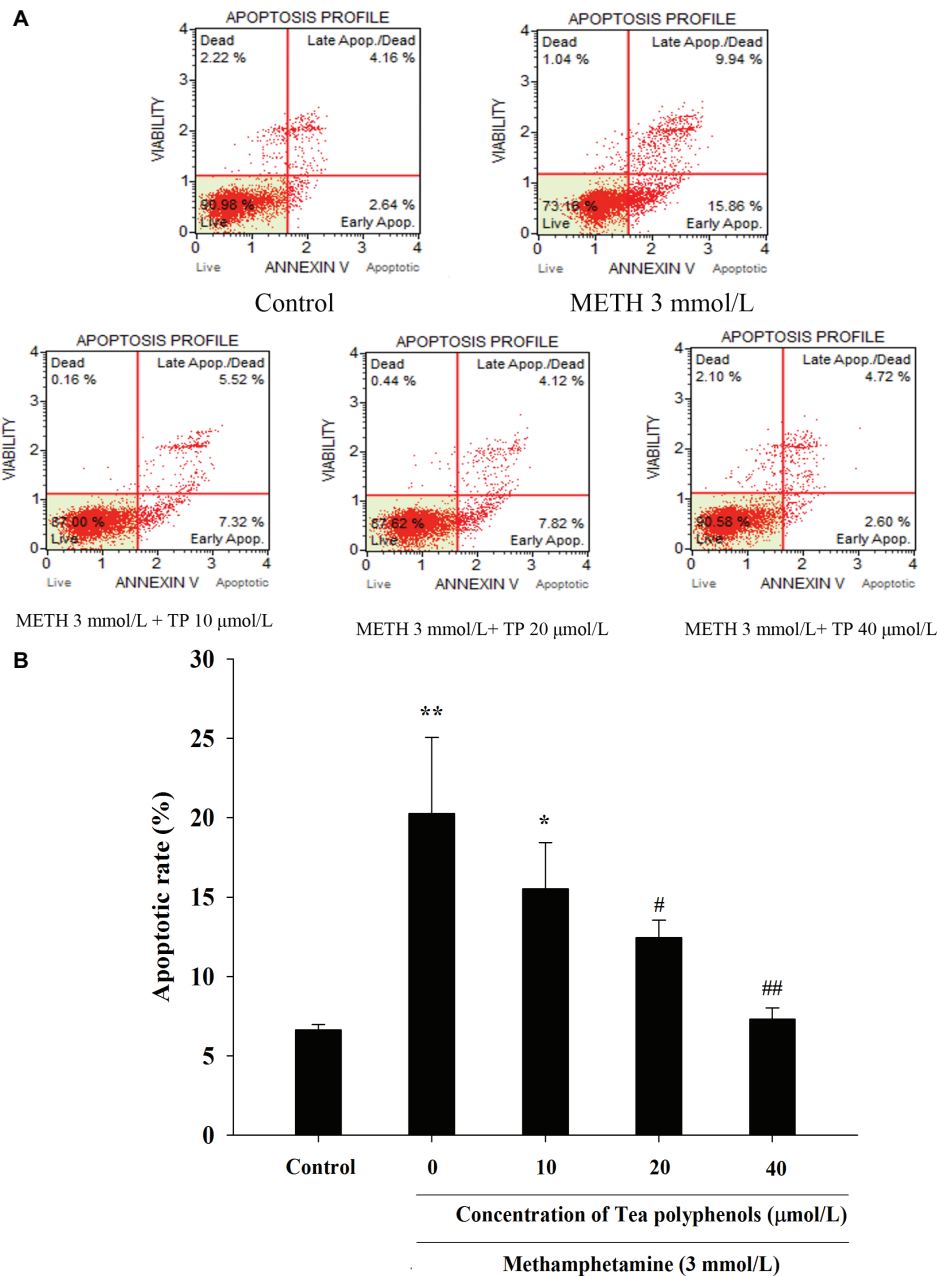
Effect of tea polyphenols on apoptosis in PC12 cells after METH exposure. PC12 cells were treated with METH (3.0 mmol/L) with or without treatment with tea polyphenols (10, 20, and 40 μmol/L) for 24 h. Cell apoptosis was analyzed by Muse Annexin V & Dead Cell Assay. Representative dot plots in the live, dead, late apoptotic/dead, and early apoptotic phases are shown in the upper panel **(A)**, and the mean percentage of cell apoptosis is expressed in a histogram in the lower panel **(B)**. Data are presented as mean ± SEM; ^*^p < 0.05 and ^**^p < 0.01 versus the control group; ^#^*p* < 0.05 and ^##^*p* < 0.01 versus the METH group.

METH caused a remarkable increase in the apoptosis rate compared with the control group (*p* < 0.01). Treatments of 20 and 40 μmol/L tea polyphenols greatly decreased the apoptotic rates compared with the METH group (Figure 8, *p* < 0.05).

### Effect of Tea Polyphenols on DNA Damage After METH Exposure in PC12 Cells

**Figure 9:**
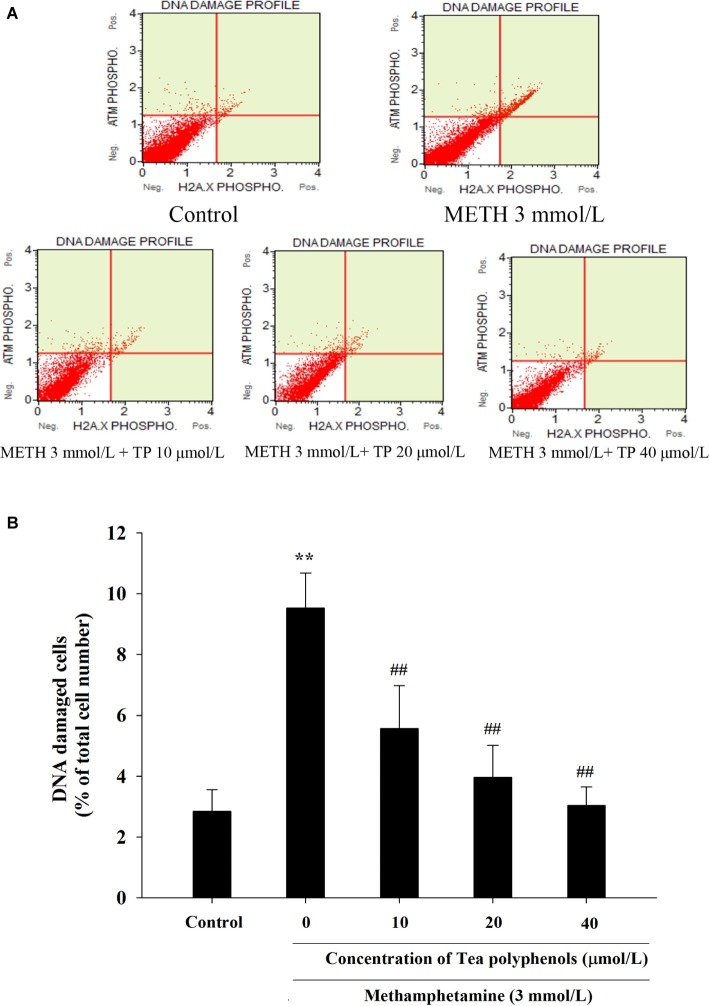
Effect of tea polyphenols on DNA damage in PC12 cells after METH exposure. PC12 cells were treated with METH (3.0 mmol/L) with or without treatment with tea polyphenols (10, 20, and 40 μmol/L) for 24 h. Cell DNA damage was analyzed by Muse Multi-Color DNA damage assay. Representative dot plots are shown in the upper panel **(A)**, and the mean percentage of DNA damaged cells is expressed in a histogram in the lower panel **(B)**. Data are presented as mean ± SEM; ^**^p < 0.01 versus the control group; ^##^*p* < 0.01 versus the METH group.

**Figure 10:**
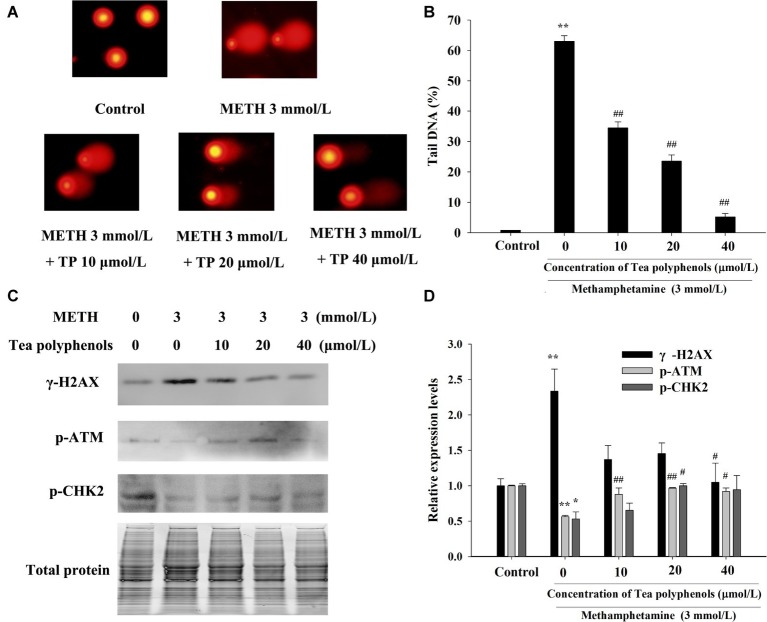
Effect of tea polyphenols on DNA damage and related protein expression in PC12 cells after METH exposure. PC12 cells were treated with METH (3.0 mmol/L) with or without treatment with tea polyphenols (10, 20, and 40 μmol/L) for 24 h. Cell DNA damage was analyzed by comet assay **(A)**, and the statistical result is expressed by a histogram in the right panel **(B)**. The expressions of γ-H2AX, p-ATM, and p-CHK2 were detected using Western Blot **(C)**; total protein was used as a loading control. Densitometric analysis of the blots is shown in **(D)**. Data are presented as mean ± SEM; ^*^p < 0.05 and ^**^p < 0.01 versus the control group; ^#^*p* < 0.05 and ^##^*p* < 0.01 versus the METH group.

Immunostaining combined with flow cytometry, comet assay, and Western Blot were performed after exposure of METH with or without tea polyphenols to investigate the protection provided by tea polyphenols against DNA damage in PC12 cells. After METH exposure, the percentage of DNA-damaged cells increased compared with the control group (Figure 9). All concentrations of tea polyphenols could remarkably reduce the percentage of DNA-damaged cells (*p* < 0.01). The results of comet assay showed that the METH exposure group had a higher percentage of tail DNA than the control group (Figures 10A,B, *p* < 0.01). The METH-induced DNA damage was remarkably decreased in the tea polyphenols pretreatment groups (*p* < 0.05). Furthermore, the protein expression of γ-H2AX in the METH group was substantially higher than that in the control group (*p* < 0.01). In contrast, 40 μmol/L tea polyphenols significantly decreased protein expression of γ-H2AX (*p* < 0.05).

Additionally, the protein expressions of p-Chk2 and p-ATM were significantly decreased after 3 mmol/L METH treatment (*p* < 0.05). In contrast, 20 μmol/L tea polyphenols significantly increased the protein levels of p-ATM and p-Chk2 (Figures 10C,D, *p* < 0.05). Therefore, the above findings indicated that tea polyphenols could reduce METH-induced DNA damage by increasing the expressions of DNA repair-related proteins in PC12 cells.

## Discussion

METH is a widely abused central neurostimulant that has been shown to produce complex neurotoxicity (Du et al., 2018; Li et al., 2018). The exact mechanism of the toxic effects of METH has not been fully elucidated, despite increasing evidence regarding the nerve cell damage induced by METH. Moreover, there is still a lack of effective treatment strategies for the neurotoxicity caused by METH, and more effective candidates need to be developed. In the present study, levels of NO and ROS were significantly increased after METH treatment *in vitro*. We also found that DNA damage and apoptosis were triggered by METH in PC12 cells, while tea polyphenols could alleviate METH-induced DNA damage and apoptosis *via* increasing antioxidant capacity and the expressions of DNA damage repair-associated proteins. These findings indicated that METH can cause a significant increase in free radicals and induce DNA damage and cell apoptosis and that this can be reversed by tea polyphenols.

Mounting evidence suggests that the mass formation of free radicals and oxidative stress may be involved in the neurotoxicity induced by METH (Huang et al., 2017; Yang et al., 2018), although the exact underlying mechanism is not yet clear. Oxidative stress induced by METH can cause damage to proteins, lipids, and DNA, altering cellular signal transduction (Krasnova and Cadet, 2009; Shokrzadeh et al., 2015). In line with these findings, we found that METH could significantly increase levels of NO and that the increase was positively correlated with METH exposure time and concentration. The induced free radicals may be the triggering factors to induce DNA strand breaks and mitochondrial-mediated apoptosis (Chen et al., 2017). We found that the level of γ-H2AX, an indicator of DNA strand breaks, was remarkably elevated after METH treatment, and levels of γ-H2AX were also positively correlated with METH exposure time and concentration. Previous reports have shown that METH could significantly increase the apoptosis rate and elevate the protein levels of cleaved PARP, cleaved caspase-3, and Bax (Li et al., 2018; Sharikova et al., 2018; Zhao et al., 2018). In agreement with these findings, the level of cleaved caspase-3 was increased significantly after METH treatment, and METH remarkably increased depolarization of the mitochondria and the cell apoptotic rate compared with the control group. Our data, combined with previous reports, indicate that highly active free radicals and oxidative damage may be partially involved in the apoptosis caused by METH.

Unless an effective repair mechanism corrects the damage to the double helix, DNA damage may cause persistent abnormalities after mitosis and in irreplaceable cells such as neurons (Milanese et al., 2018). Fortunately, cells have evolved DNA damage repair (DDR) mechanisms to alleviate a variety of damages (Henssen et al., 2017). Once DNA damage is triggered by exogenous and endogenous factors such as free radicals, the DDR can be activated to alter expressions of the damage sensor γ-H2AX and subsequent signal transduction pathways such as ATM/Chk2 pathway-related proteins (Ronco et al., 2017; He et al., 2018). If the damage is mild, it can be repaired through DDR; otherwise, it will result in gene mutation or apoptosis (Jackson and Bartek, 2009). However, whether DNA damage and repair-related proteins are involved in the neurotoxicity induced by METH remains unclear. In the present study, the expressions of p-ATM and p-Chk2 were significantly increased at 3 h after METH treatment and were reduced at 24 h. Consequently, we speculated that PC12 cells excited the expressions of DDR-associated proteins as a stress defense mechanism to prevent cytotoxicity at the early phase after METH treatment. However, if DNA damage is not repaired, the cellular protective effect may not overcome the toxicity induced by METH, and cells are likely to undergo programmed cell death such as mitochondria-mediated apoptosis.

**Figure 11:**
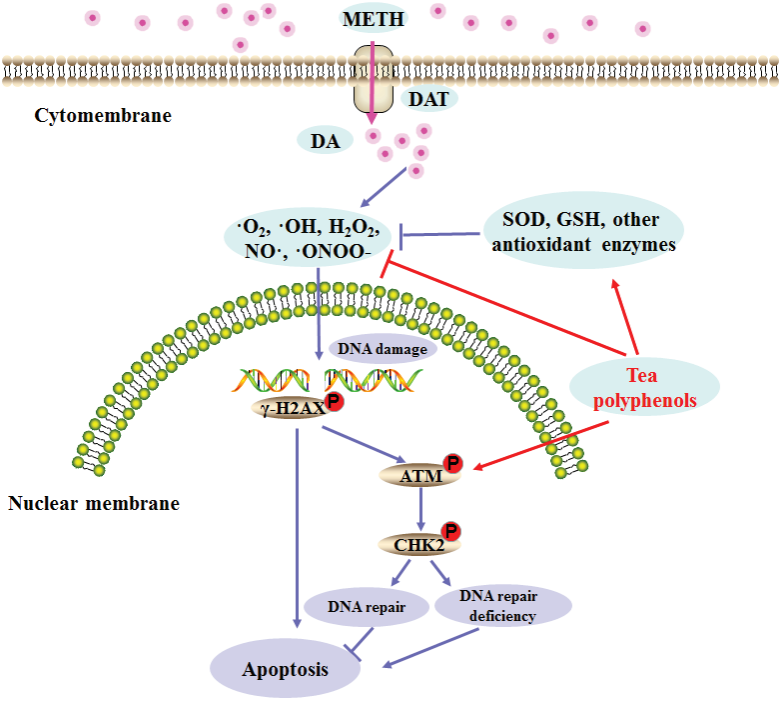
Schematic representation depicting the role of DNA damage and repair signaling pathway in METH-induced neurotoxicity.

Previous studies have found that pretreatment with antioxidants such as N-acetylcysteine and ascorbic acid can prevent METH-induced cell damage, and these reports further confirm the potential role of oxidation mechanisms in METH neurotoxicity (Chandramani Shivalingappa et al., 2012; Huang et al., 2017; Zeng et al., 2018). Tea polyphenols are bioactive catechins that have been shown to exert protection against neuronal cell damage (Ding et al., 2017; Chen et al., 2018). For example, tea polyphenols can suppress the ROS release and reduction of SOD activities and apoptosis induced by glutamate in primary cortical neurons (Cong et al., 2016). Furthermore, tea polyphenols also possess neuroprotective activities *via* the activation of the Keap1/Nrf2 pathway *in vitro* and *in vivo* (Qi et al., 2017b). In this study, we found that tea polyphenols have protective effects against METH-induced toxicity. Similar to previous studies, tea polyphenols were able to reverse the decline of SOD and GSH significantly and inhibit the increase in MDA contents as well as the production of NO and ROS that is induced by METH exposure. We further verified that tea polyphenols are highly likely to reduce the apoptosis in PC12 cells induced by METH through the mitochondria-mediated pathway. Additionally, our results indicated that tea polyphenols increased levels of DDR related proteins (p-ATM and p-Chk2) and decreased METH-induced DNA damage marker γ-H2AX expression. Based on these results, a series of events might occur in the procedure of the apoptotic pathway, and we speculated that tea polyphenols may attenuate ROS and NO production, promote the expressions of the oxidative enzyme system and DDR-related proteins, protect against DNA damage, and prevent apoptosis during treatment with METH. A schematic representation presenting the relationship among oxidative stress, DNA damage, and apoptosis after METH treatment and the underlying mechanism of action of tea polyphenols on the apoptosis induced by METH is shown in Figure 11.

In summary, oxidative stress, DNA damage, and apoptosis are interrelated in the pathology of many nerve system diseases. In the current study, we found that tea polyphenols protected against the neurotoxicity induced by METH in PC12 cells. Furthermore, we have demonstrated that the protective effect of tea polyphenols was mediated through attenuated oxidative stress, DNA damage, and mitochondrial apoptosis. Therefore, our research supports the hypothesis that supplementation with tea polyphenols might effectively prevent METH-induced neurotoxicity and neurodegenerative disease, and it is necessary to carry out further investigation in the future.

## Data Availability Statement

All datasets generated for this study are included in the article/supplementary material.

## Author Contributions

QR and CL designed the study. QR, QX, and XT processed the MTT assay, LDH assay, apoptosis detection, oxidative stress, and DNA damage tests. LC and MZ collected and analyzed data. YL interpreted the data. QR wrote and edited the manuscript. All authors critically reviewed the content and approved the final version for publication.

### Conflict of Interest

The authors declare that the research was conducted in the absence of any commercial or financial relationships that could be construed as a potential conflict of interest.
